# The role of Zur-regulated lipoprotein A in bacterial morphology, antimicrobial susceptibility, and production of outer membrane vesicles in *Acinetobacter baumannii*

**DOI:** 10.1186/s12866-020-02083-0

**Published:** 2021-01-18

**Authors:** Nayeong Kim, Hyo Jeong Kim, Man Hwan Oh, Se Yeon Kim, Mi Hyun Kim, Joo Hee Son, Seung Il Kim, Minsang Shin, Yoo Chul Lee, Je Chul Lee

**Affiliations:** 1 Department of Microbiology, School of Medicine, Kyungpook National University, 680 Gukchaebosang-ro, Jung-gu, Daegu, 41944 Republic of Korea; 2 Department of Nanobiomedical Science, Dankook University, Cheonan, South Korea; 3 Drug & Disease Target Team, Korea Basic Science Institute, Ochang, South Korea; 4 Department of Bio-Analytical Science, University of Science and Technology (UST), Daejeon, South Korea

**Keywords:** *Acinetobacter baumannii*, Zur-regulated gene, ZrlA, Carboxypeptidase, Outer membrane vesicle

## Abstract

**Background:**

Zinc uptake-regulator (Zur)-regulated lipoprotein A (ZrlA) plays a role in bacterial fitness and overcoming antimicrobial exposure in *Acinetobacter baumannii*. This study further characterized the *zrlA* gene and its encoded protein and investigated the roles of the *zrlA* gene in bacterial morphology, antimicrobial susceptibility, and production of outer membrane vesicles (OMVs) in *A. baumannii* ATCC 17978.

**Results:**

In silico and polymerase chain reaction analyses showed that the *zrlA* gene was conserved among *A. baumannii* strains with 97–100% sequence homology. Recombinant ZrlA protein exhibited a specific enzymatic activity of D-alanine-D-alanine carboxypeptidase. Wild-type *A. baumannii* exhibited more morphological heterogeneity than a Δ*zrlA* mutant strain during stationary phase. The Δ*zrlA* mutant strain was more susceptible to gentamicin than the wild-type strain. Sizes and protein profiles of OMVs were similar between the wild-type and Δ*zrlA* mutant strains, but the Δ*zrlA* mutant strain produced 9.7 times more OMV particles than the wild-type strain. OMVs from the Δ*zrlA* mutant were more cytotoxic in cultured epithelial cells than OMVs from the wild-type strain.

**Conclusions:**

The present study demonstrated that *A. baumannii* ZrlA contributes to bacterial morphogenesis and antimicrobial resistance, but its deletion increases OMV production and OMV-mediated host cell cytotoxicity.

**Supplementary Information:**

The online version contains supplementary material available at 10.1186/s12866-020-02083-0.

## Background

*Acinetobacter baumannii* is a leading cause of nosocomial infections, including ventilator-associated pneumonia, skin and soft tissue infections, urinary tract infections, meningitis, and sepsis, particularly in intensive care units [1, 2]. *A. baumannii* is a member of the ‘ESKAPE’ pathogens that are potentially drug-resistant bacteria [3]. The World Health Organization listed carbapenem-resistant *A. baumannii* as the most critical pathogen for development of new therapeutic agents. Like other pathogens, *A. baumannii* acquires nutrient metals, including iron, zinc (Zn), copper, magnesium, nickel, and manganese, from the host for a variety of biological processes [4–6]. However, hosts can limit the availability of these metals in a process referred to as nutritional immunity [4]. The acquisition of Zn and its utilization are associated with pathogenesis in *A. baumannii* [7]. The Zn uptake-regulator (Zur) is a conserved repressor that controls the expression of Zur-regulated genes. Mortensen et al. [7] identified 144 genes that were significantly up-regulated or down-regulated in expression in the Δ*zur*::Km mutant compared to that in *A. baumannii* ATCC 17978 using RNA-sequencing analysis [7]. The *A1S_3412* gene encoding Zur-regulated lipoprotein A (ZrlA) is a significantly up-regulated (18.8-fold) during Zn starvation [7]. An Δ*zrlA* mutant exhibits increased envelope permeability and decreased membrane barrier function, which subsequently increases susceptibility to antimicrobial agents [8]. Moreover, this mutant strain exhibits reduced biofilm formation and surface motility, low adherence to epithelial cells, and low bacterial burden in the bloodstream compared to wild-type [9]. Thus, ZrlA contributes to antimicrobial resistance and pathogenicity in *A. baumannii* and is a potential target for anti-virulence agents against multidrug-resistant *A. baumannii*.

All bacterial cells, including gram-positive and gram-negative bacteria, produce extracellular vesicles (EVs) [10–12]. Bacteria-derived EVs are involved in biological processes such as nutrient acquisition, biofilm formation, horizontal gene transfer, and cell to cell communication [10, 13–18]. Also, bacterial EVs contribute to pathogenic events in host-pathogen interactions regarding the delivery of virulence factors and toxins, host cell death, and inflammatory responses [13, 19–21]. Little is known about mechanisms of EV biogenesis for gram-positive bacteria, whereas several models for outer membrane vesicle (OMV) biogenesis in gram-negative bacteria have been proposed, including a reduction in crosslinking between peptidoglycans and the outer membrane [21–24], deacylation of lipopolysaccharides [25], accumulation of phospholipids in the outer leaflet of outer membranes [18, 26], and localized membrane remodeling [27], suggesting that OMVs are likely to be produced by several pathways in gram-negative bacteria. OMV production is stimulated by harsh environments, such as presence of antimicrobial agents, and envelope and oxidative stresses [13, 14, 28]. Further, sequestration of divalent cations such as Mg^2+^ and Ca^2+^ increases OMV production [18]. However, the effect of Zn or Zur-regulated genes on OMV biogenesis has not been determined.

In this study, we explored the hypothesis that ZrlA plays a role in bacterial morphogenesis and OMV biogenesis, because ZrlA possesses peptidase activity for peptidoglycan remodeling [8], which may affect the crosslinking between peptidoglycans and outer membranes. Moreover, we further characterized the *zrlA* gene and its encoded protein, even though ZrlA is known to be a Zn-binding peptidase located in the inner membrane [8].

## Results

### Characterization of the *zrlA* gene and its encoded protein

**Fig. 1:**
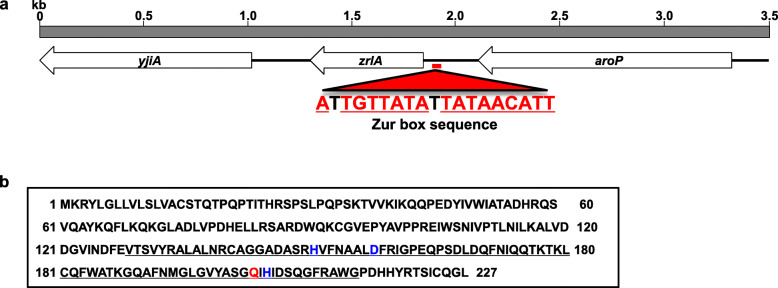
Physical map and sequence analysis of the *zrlA* gene in *A. baumannii* ATCC 17978. a Arrows indicate coding regions of *yjiA*, *zrlA*, and *aroP*. The figure was generated from the nucleotide sequence of *A. baumannii* (GenBank Accession No. NZ_CP018664.1). Predicted Zur box sequences are indicated in red. b Analysis of the predicted amino acid sequence of ZrlA using the MEROPS database (https://www.ebi.ac.uk/merops/). The peptidase domain is indicated with solid underlining. The active site (red) and metal ion ligands (blue) are also indicated

The complete sequence of the *zrlA* gene and surrounding genes in *A. baumannii* ATCC 17978 was analyzed (GenBank accession number NZ_CP018664.1). The *zrlA* gene (*A1S_3412*) is 684 bp long, and it is predicted to encode 227-amino acid protein. Two adjacent genes, *yjiA* (*A1S_3411*) and *aroP* (*A1S_3413*), were predicted to encode a putative GTPase and an APC family aromatic amino acid transporter, respectively (Fig. 1a). Sequence analysis showed a palindromic Zur box sequence in the promoter region of the *zrlA* gene. The *zrlA* gene was predicted to encode a peptidase of the M15 family (https://www.ebi.ac.uk/merops/), with an 86 residue peptidase domain between amino acids 129–214 (Fig. 1b). In enterococci, VanX is known to be a Zn-dependent D-alanine-D-alanine carboxypeptidase (DD-CPase) with H116, D123, and H184 being Zn-coordinated residues [29]. Three metal ligands (H150, D157, and H204) and an active site residue (Q202) were also present in the peptidase domain of ZrlA. These motifs, HXXXXXXD and QXH, were similar to the motifs HXXXXXXD and WXH found in peptidoglycan hydrolase of *Burkholderia pseudomallei* phage ST79 [30]. Sequence analysis indicated that the *zrlA* gene was conserved in all sequenced *A. baumannii* strains with 97–100% homology (https://blast.ncbi.nlm.nih.gov/). All amino acid variations were located outside of peptidase domains. Moreover, the *zrlA* gene was amplified in all tested clinical *A. baumannii* isolates from Korean hospitals, as well as in ATCC 19606^T^ (Supplementary Fig. 1). Next, a recombinant ZrlA protein was expressed in *Escherichia coli* BL21 (Supplementary Fig. 2), and the enzymatic activity of recombinant ZrlA protein was assessed by the fluorimetric *o*-phthaldialdehyde (OPTA) method. The recombinant protein specifically cleaved the terminal D-alanine of N_α_,N_ε_-diacetyl-L-Lys-D-Ala-D-Ala, but its enzymatic activity was lower for acetyl-L-Lys-D-Ala-D-Ala and D-Ala-D-Ala (Table 1), suggesting specific DD-CPase activity.

**Table 1:** DD-CPase activity of the recombinant ZrlA protein

Substrate	Enzymatic activity of rZrlA protein (U/mg)^a^
D-Ala-D-Ala	9.6 ± 4.4
Acetyl-L-Lys-D-Ala-D-Ala	6.5 ± 4.2
N_α_,N_ε_-diacetyl-L-Lys-D-Ala-D-Ala	34.9 ± 3.8

^a^Enzymatic activity (U/mg protein) of recombinant ZrlA proteins was determined using the fluorimetric *o*-phthaldialdehyde method. Recombinant ZrlA protein (40 μg) was added to 10 mM solutions of indicated substrates. The results are the mean ± SD. Assays were performed in triplicate

### Role of ZrlA in bacterial morphology and antimicrobial susceptibility

**Fig. 2:**
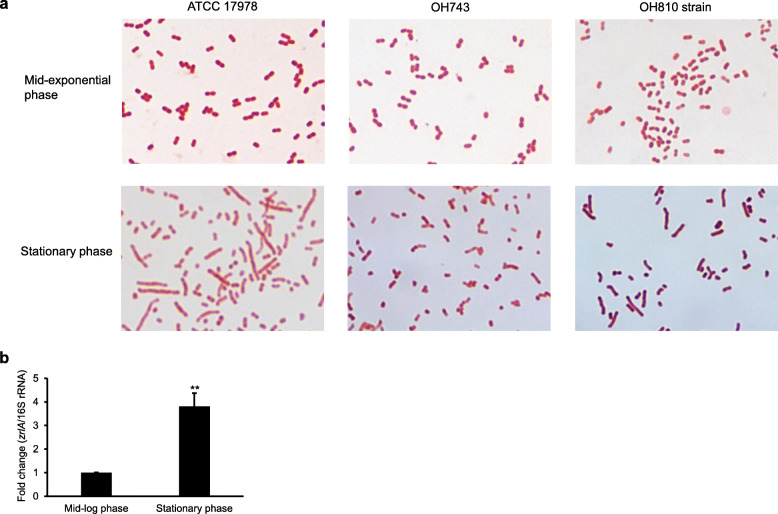
Morphological characteristics of the Δ*zrlA* mutant strain. **a** *A. baumannii* ATCC 17978, Δ*zrlA* mutant OH743, and *zrlA*-complemented OH810 strains were cultured in LB to mid-exponential or stationary phases, and cellular morphology was visualized after Gram staining. Magnification: 1000×. **b** Transcription levels of *zrlA* in *A*. *baumannii* ATCC 17978 were determined using qPCR. Data are presented as mean ± SD of three independent experiments. ** *p* < 0.01 comparing the expression of *zrlA* at mid-log phase

We investigated morphological differences between wild-type and Δ*zrlA* mutant OH743 strains because DD-CPases contribute to cell separation and peptidoglycan remodeling [31]. The wild-type and Δ*zrlA* mutant strains appeared as gram-negative coccobacilli at mid-exponential phase and no morphological difference was observed between the wild-type and OH743 strains at mid-exponential phase (Fig. 2a). The wild-type strain showed more morphological heterogeneity than the Δ*zrlA* mutant strain at stationary phase. Bacterial morphology of the *zrlA*-complemented OH810 strain was partially restored compared with that of the wild-type strain. Expression of the *zrlA* gene in *A. baumannii* ATCC 17978 was higher during stationary phase than during exponential phase (Fig. 2b). Minimum inhibitory concentrations (MICs) of 15 antimicrobial agents for the wild-type, OH743, and OH810 strains were determined to address the effects of Δ*zrlA* mutation on antimicrobial susceptibility. Gentamicin showed a 4-fold decrease in MICs for the OH743 strain, and colistin, tobramycin, and erythromycin showed a 2-fold decrease in MICs for the OH743 strain (Table 2). MICs for the remaining antimicrobial agents for the OH743 strain were the same or similar (< 2-fold change) to MICs for the wild-type strain. These results suggest that ZrlA contributes to bacterial morphogenesis and moderate resistance to several antimicrobial agents.

**Table 2:** MICs of antimicrobial agents for *A. baumannii* strains used in this study

Antimicrobial agent	MIC (μg/ml)		
	ATCC 17978	OH743	OH810
Aztreonam^a^	24	24	24
Ceftazidime^a^	3	3	3
Cefoxitin^b^	128	128	128
Imipenem^a^	0.19	0.19	0.19
Meropenem^b^	0.5	0.5	0.5
Colistin^b^	0.25	0.125	0.25
Ciprofloxacin^a^	0.125	0.19	0.125
Levofloxacin^b^	0.125	0.125	0.125
Nalidixic acid^a^	4	3	3
Gentamicin^a^	0.5	0.125	0.25
Tobramycin^b^	0.5	0.25	0.5
Tetracycline^a^	1.5	1.5	1.5
Tigecycline^b^	0.125	0.094	0.125
Trimethoprim^a^	> 32	> 32	> 32
Erythromycin^b^	16	8	16

^a^ The MICs were determined by the Etest method

^b^ The MICs were determined by broth microdilution

### Role of *zrlA* in the production of OMVs

**Fig. 3:**
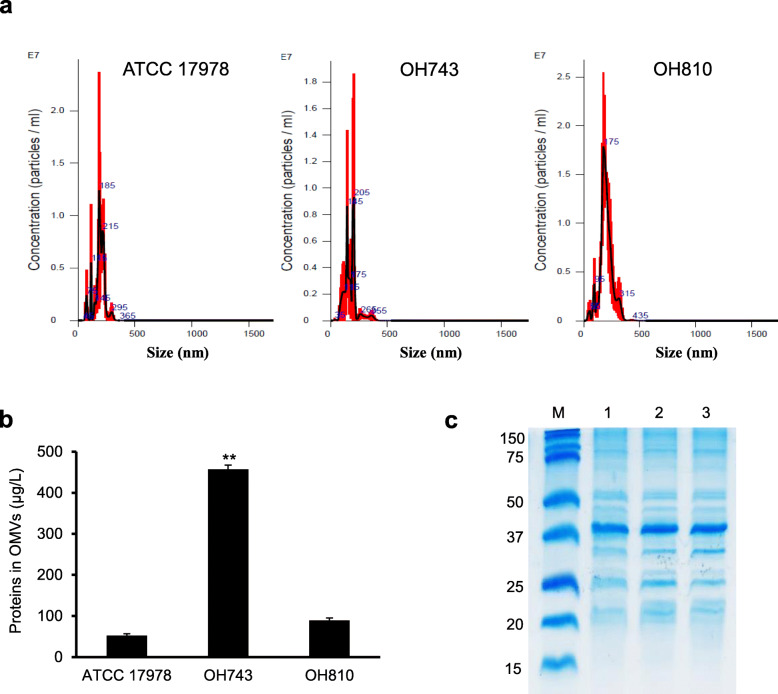
OMVs of *A. baumannii* ATCC 17978, Δ*zrlA* mutant OH743, and *zrlA*-complemented OH810 strains **a** Size and number of OMVs from *A. baumannii* strains were determined using NTA. Data are representative of three independent experiments with similar results. **b** Protein concentration of OMVs isolated from 1 L of bacterial culture was measured. Data are presented as mean ± SD of two independent experiments. ** *p* < 0.01 compared to wild-type ATCC 17978. **c** OMV proteins were resolved by SDS-PAGE in 12% gels. Lane M, molecular weight marker; 1, *A. baumannii* ATCC 17978; 2, Δ*zrlA* mutant OH743; 3, Δ*zrlA*-complemented OH810

Bacteria were cultured in lysogeny broth (LB) and OMVs were isolated from culture supernatants. Sizes of OMVs from the wild-type, OH743, and OH810 strains were 197.8 ± 16.0 nm, 180.9 ± 25.9 nm, and 190.7 ± 18.2 nm, respectively, using nanoparticle tracking analysis (NTA) (Fig. 3a). OMV particles from 1 L culture of the wild-type, OH743, and OH810 strains contained 2.96 × 10^12^, 2.87 × 10^13^, and 2.58 × 10^12^ particles, respectively. The OH743 strain produced more OMV proteins (457.1 ± 10.5 μg/L) than the wild-type strain (52.1 ± 4.6 μg/L) (Fig. 3b). The OH743 strain produced 9.7 times more OMV particles and 8.8 times more OMV proteins than the wild-type strain, but the OH743 strain produced small sizes of OMVs as compared to the wild-type strain. Sodium-dodecyl sulfate-polyacrylamide gel electrophoresis (SDS-PAGE) analysis showed similar protein profiles among the three OMV samples (Fig. 3c). These results suggest that ZrlA negatively affects OMV production in *A. baumannii*.

### Effect of *zrlA* on OMV-mediated cytotoxicity in epithelial cells

**Fig. 4:**
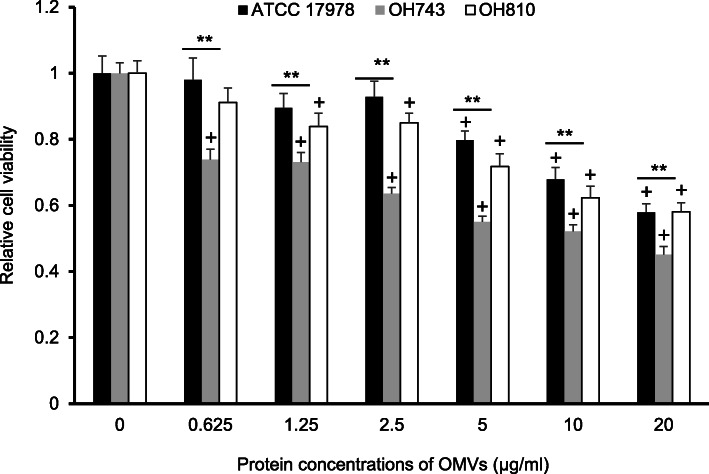
Host cell cytotoxicity induced by *A. baumannii* OMVs. A549 cells were treated with OMVs from *A. baumannii* strains for 24 h and then cell viability was determined using the MTT assay. Data are presented as mean ± SD of three independent experiments. + *p* < 0.01 compared to untreated control cells. ** *p* < 0.01 comparing the same concentration of OMVs from *A. baumannii* ATCC 17978

A549 cells were incubated with *A. baumannii* OMVs for 24 h and then cell viability was determined using the 3-[4,5-dimethylthiazol-2-yl]-2,5 diphenyltetrazolium bromide (MTT) assay. The wild-type strain OMVs triggered cytotoxicity at 5 μg/ml, whereas the Δ*zrlA* mutant OMVs triggered cytotoxicity at ≤0.625 μg/ml (Fig. 4). Cytotoxicity was significantly different between wild-type and OH743 mutant OMVs at concentrations ≥0.625 μg/ml. These results suggest that the *zrlA* gene negatively affects host cell cytotoxicity induced by *A. baumannii* OMVs.

## Discussion

The *zrlA* gene is known to encode a Zn-binding DD-CPase located in the inner membrane [8]. Still, its presence and genetic variability among *A. baumannii* strains are not yet characterized. In silico and polymerase chain reaction (PCR) analyses indicated that the *zrlA* gene was conserved among *A. baumannii* strains with high sequence homology. Peptidase motifs typically found in DD-CPases were found in the predicted protein encoded by the *zrlA* gene. Recombinant ZrlA proteins specifically cleaved the terminal D-alanine of N_α_,N_ε_-diacetyl-L-Lys-D-Ala-D-Ala, a substrate for penicillin-sensitive D-alanine CPase. Zur-regulated *zrlA* gene is thus conserved in *A. baumannii* strains, and its encoding protein ZrlA possesses a specific DD-CPase activity.

DD-CPase, a member of the penicillin-binding proteins (PBPs), cleaves the terminal D-alanine from a muramyl pentapeptide. PBPs are classified into high molecular mass (HMM) and low molecular mass (LMM) PBPs based on amino acid sequence, molecular weight, and enzymatic activity [31–33]. HMM PBPs are responsible for polymerization of peptidoglycan and inter-strand crosslinking of adjacent peptidoglycan molecules [31, 34, 35]. HMM PBPs play a role in bacterial cell elongation, maintenance of cell morphology, and cell division. LMM PBPs, including DD-CPases and endopeptidases, contribute to cell separation and peptidoglycan remodeling. LMM PBPs are not essential for bacterial growth. The structure and function of HMM PBPs are well studied, but biological functions of LMM PBPs remain poorly investigated. In limited studies of *A. baumannii,* a LMM PBP 7/8, a D-alanine-D-alanine endopeptidase, was critical for survival in vitro and in vivo and contributed to serum resistance [36]. A deletion mutant of the PBP 7/8 gene in *A. baumannii* showed more coccobacillary forms than wild-type. Deletion of PBP7/8 seems to alter the structure of peptidoglycan, which in turn affects cell morphology and survival of *A. baumannii* both in vitro and in vivo. In the previous study, bacterial growth was not different between wild-type and Δ*zrlA* mutant strains cultured under shaking and static conditions [9], indicating that the *zrlA* gene is not essential for bacterial survival and growth. However, in the present study, wild-type *A. baumannii* grown to stationary phase showed more morphological heterogeneity than the Δ*zrlA* mutant strain. Morphological difference was not observed between wild-type and Δ*zrlA* mutant strains at mid-exponential phase, in contrast to a previous study that demonstrated more morphological heterogeneity in wild-type than Δ*zrlA* mutant strains cultured to mid- to late-exponential phase under Zn-replete conditions [8]. The expression of the *zrlA* gene was higher in sessile cells than planktonic cells [9]. Moreover, morphological heterogeneity is correlated with expression level of the *zrlA* gene among growth phases. ZrlA contributes to overcoming sub-MICs of antibiotic exposure in vitro and in vivo [8]. The Δ*zrlA* mutant exhibits increased envelope permeability. Mutant cells show increased susceptibility to antibiotics, including carbenicillin, vancomycin, tetracycline, and polymyxins B, and detergents, including SDS and ethylenediaminetetraacetic acid, in vitro compared to the wild-type strain [8]. The present study also showed that the Δ*zrlA* mutant was more susceptible to gentamicin, colistin, tobramycin, and erythromycin than the wild-type strain, but MICs were not greatly different between the two strains. Our results suggest that ZrlA is an LMM PBP possessing a specific DD-CPase activity involved in morphological plasticity and moderate antimicrobial resistance.

The production of OMVs increases in response to nutrient restriction and exposure to antibiotics or chemicals [13, 14, 28]. A reduction in crosslinking between the outer membrane and peptidoglycan also increases OMV production [21–24]. OmpA via the C-terminal OmpA-like domain interacts with diaminopimelate of peptidoglycans [37] and a Δ*ompA* mutant of *A. baumannii* produced more OMVs than the wild-type strain [38]. In *E. coli*, peptidoglycans are covalently crosslinked to the outer membrane via short outer membrane-anchored lipoprotein Lpp. Levels of Lpp crosslinking to peptidoglycan negatively correlate with OMV production [23, 39]. Spr is a murein DD-endopeptidase located in the outer membrane of *E. coli*. Δ*spr* mutants inhibit peptidoglycan turnover and other PBPs, such as PBP4, induce compensatory increases in peptidoglycan synthesis. This increase of peptidoglycans reduces the ability to form sufficient Lpp-outer membrane crosslinks [40]. This mutation may result in four times more OMV production in Δ*spr* mutant than wild-type. The equilibrium between peptidoglycan synthesis and degradation may affect OMV production by altering the numbers of covalently crosslinked Lpp to peptidoglycan. In the present study, the Δ*zrlA* mutant produced 9.7-fold more OMV particles than the wild-type strain. However, sizes of OMVs from the Δ*zrlA* mutant were smaller than those from the wild-type strain. Regarding the number of OMV particles and their protein concentrations, each OMV particle from the Δ*zrlA* mutant strain may carry slightly less proteins than OMVs from the wild-type strain. ZrlA in *A. baumannii* displays peptidase activity like Spr in *E. coli*. Hence, the Δ*zrlA* mutant may inhibit peptidoglycan remodeling and decrease interactions between outer membrane proteins and peptidoglycans, resulting in increased envelope permeability and hyperproduction of OMVs.

Stress-inducing conditions alter both production and molecular composition of OMVs [28, 38]. Alterations in lipopolysaccharides, proteins, peptidoglycans, and pathogen-associated molecular patterns of OMVs likely trigger different host cell responses. OMVs from a Δ*bfmS* mutant were more cytotoxic in A549 cells than OMVs from the wild-type strain [41], yet OMVs from the Δ*ompA* mutant were less cytotoxic than OMVs from the wild-type strain [19]. The present study showed that OMVs from the Δ*zrlA* mutant were more cytotoxic in A549 cells than the wild-type strain. OmpA was identified as a cytotoxic factor packaged in *A. baumannii* OMVs [19]. SDS-PAGE analysis of OMVs revealed that protein profiles were similar between the wild-type and Δ*zrlA* mutant strains. Even though SDS-PAGE images show similar profiles, differences in protein content between OMVs from the wild-type and Δ*zrlA* mutant strains cannot be excluded. SDS-PAGE analysis is less appropriate than two-dimensional gel electrophoresis. This study did not identify difference in protein content and cytotoxic factors in the Δ*zrlA* mutant OMVs, an issue that should be investigated in further studies.

## Conclusions

The present study demonstrates the interplay between ZrlA, peptidoglycan dynamics, bacterial morphogenesis, and OMV production in *A. baumannii*. ZrlA contributes to overcoming antibiotic exposure and augments pathogenicity of *A. baumannii* both in vitro and in vivo [7–9]. The *zrlA* gene or its protein is a possible therapeutic target for treating *A. baumannii* infection. However, deletion of the *zrlA* gene increased the OMV production in *A. baumannii*. Moreover, OMVs produced by the Δ*zrlA* mutant were more cytotoxic to epithelial cells than OMVs from the wild-type strain. These observations provide opposing perspectives of ZrlA for anti-virulence strategies against *A. baumannii* [42].

## Methods

### Bacterial strains

*A. baumannii* ATCC 17978, Δ*zrlA* mutant OH743 strain (ATCC 17978 with Δ*zrlA*), and *zrlA*-complemented OH810 strain (OH743 with *zrlA* in chromosome) were used in this study [9]. Ten clinical *A. baumannii* isolates were obtained from the Kyungpook National University Hospital Culture Collection for Pathogens (KNUH-CCP). *E. coli* BL21 (DE3) star cells were used for production of recombinant ZrlA proteins. Bacteria were cultured in LB or blood agar plates at 37 °C.

### Detection and cloning of the *zrlA* gene and recombinant protein production

PCR was performed to detect the *zrlA* gene using primers A1 (5′-GCT TTT ATA GTC CCT GAC A-3′) and A2 (5′-CTG TGG TTA AAA TCA AAC AA-3′). Genomic DNA was purified from *A*. *baumannii* strains using the SolGent™ Genomic DNA prep kit (SolGent, Daejeon, Korea). The full-length *zrlA* gene was amplified using primers C1 (5′- GGG CGG CGG TGG TGG CGG CAT GAA GCG TTA TTT AGG TTT A-3′) and C2 (5′- GTT CTT CTC CTT TGC GCC CTA TAG TCC CTG ACA AAT TGA GG-3′), designed for ligation-independent cloning [43]. *A*. *baumannii* ATCC 17978 genomic DNA was used as the PCR template. PCR products were treated with T4 DNA polymerase (New England Biolabs, Ipswich, MA) and inserted into the ligation-independent cloning expression vector pB4, a derivative of pET21a (Novagen, Madison, WI) [44]. DNA fragments and plasmid DNA were purified using the AccuPrep Gel Purification Kit (Bioneer, Daejeon, Korea) and the AccuPrep® Plasmid Extraction Kit (Bioneer), respectively. Plasmid construct pB4:*zrlA* was transformed into *E. coli* BL21 (DE3) star cells. ZrlA protein was purified using sequential chromatographic steps as previously described [45].

### DD-CPase assays of recombinant ZrlA protein

Enzyme activity of recombinant ZrlA protein was assessed by measuring the release of D-Ala from N_α_,N_ε_-diacetyl-L-Lys-D-Ala-D-Ala, acetyl-L-Lys-D-Ala-D-Ala, and D-Ala-D-Ala (Sigma-Aldrich, St. Louis, MO) using the OPTA method as previously described [46, 47]. Fluorescence intensity (λex = 340 nm: λem = 455 nm) was measured using a fluorescence microplate reader (Tecan Spark 10 M, Austria). Enzymatic activity was quantified using a standard curve with D-Ala. One unit of DD-CPase activity was defined as the amount of enzyme that produced 1 μM of D-Ala per min [46]. Assays were performed in triplicate.

### Gram staining of bacteria

Bacteria were cultured in LB with shaking to optical density at 600 nm (OD_600_) of 1.2 (mid-exponential phase) or 1.8 (stationary phase). Bacteria were washed with phosphate-buffered saline (PBS). After centrifugation, bacterial pellets were stained with Gram’s reagents.

### RNA isolation and quantitative real time PCR

Bacteria were cultured in LB with shaking to OD_600_ of 1.2 and 1.8 for mid-exponential and stationary phases, respectively. Total RNA was extracted using a RNeasy Mini Kit (Qiagen, Valencia, CA). cDNA was synthesized by reverse transcription with 1.5 μg of total RNA using random hexamer primer and RevertAid reverse transcriptase in a total reaction volume of 20 μl (Thermo Fisher Scientific, Waltham, MA). Specific primers for the *zrlA* gene, 5′-CCC AGC CGA CGA TTA CTC AT-3′ and 5′-GCG ATC CAA ACG ACA TAA TCT TC-3′, and 16S rRNA gene, 5′-GCA CAA GCG GTG GAG CAT-3′ and 5′-CGA AGG CAC CAA TCC ATC TC-3′, were used. Gene transcripts was quantified using a Thermal Cycler Dice TP850 (Takara Bio, Shiga, Japan) with SYBR Premix Ex Taq (Takara Bio) following the manufacturer’s instructions. Amplification specificity was evaluated using melting curve analysis. Gene expression was normalized to 16S rRNA expression in each sample, and fold change was analyzed using the ΔΔCt method.

### Antimicrobial susceptibility test

The MICs of aztreonam, ceftazidime, nalidixic acid, ciprofloxacin, gentamicin, imipenem, tetracycline, and trimethoprim were determined by the Etest method following the manufacturer’s instructions (bioMe’rieux, Marcy-l’_Etoile, France). MICs of colistin, tigecycline, cefoxitin, meropenem, levofloxacin, erythromycin, and tobramycin were determined by broth microdilution following guidelines of the Clinical Laboratory Standards Institute (CLSI) [48]. *E. coli* ATCC 25922 and *Pseudomonas aeruginosa* ATCC 27853 were used as quality control strains.

### Isolation of OMVs

The OMVs derived from *A. baumannii* were purified from culture supernatants as previously described [19, 28]. In brief, bacteria were cultured in LB with shaking to reach OD_600_ of 1.5. Bacterial cells were removed by centrifugation at 8000 *g* for 20 min and then culture supernatants were filtered with a 0.22 μm membrane. The samples were concentrated using a QuixStand Benchtop System (GE Healthcare, Amersham, UK) with a 500 kDa hollow fiber membrane (GE Healthcare). OMVs were isolated by ultracentrifugation at 150,000 *g* at 4 °C for 3 h. OMVs were resuspended in PBS and protein contents were measured with a modified bicinchoninic acid (BCA) assay (Thermo Fisher Scientific). OMV proteins were separated on 12% SDS-PAGE gel and stained with Coomassie brilliant blue R-250 (Bio-Rad, Hercules, CA). The sterility of OMV samples were checked by streaking on blood agar plates.

### Nanoparticle tracking analysis

Size and number of OMVs were measured by a NanoSight NS500 instrument with 488 nm laser and sCMOS camera modules (Malvern Instruments, Worcestershire, UK) [49]. Captured data were analyzed using NTA 3.1 software build 3.1.46. Experiments were performed in triplicate.

### Cell culture and cell viability test

A549 cells derived from human lung carcinoma were obtained from the Korean Cell Line Bank (Seoul, Korea). Cells were grown in RPMI 1640 medium (HyClone, Logan, UT) supplemented with 10% heat inactivated fetal bovine serum (HyClone), 2.0 mM _L_-glutamine, and 100 U/ml penicillin at 37 °C in a 5% CO_2_. Cells were seeded at a concentration of 2 × 10^4^/well in a 96-well microplate. Cell viability was measured using an MTT assay (Sigma-Aldrich). A549 cells were treated with *A. baumannii* OMVs for 24 h and then viability of cells was determined at 600 nm 3 h after treatment with MTT reagent. Assays were performed in triplicate in three independent experiments.

### Statistical analysis

All data are presented as mean ± standard deviation (SD). Data were analyzed using R 3.6.3 (https://www.r-project.org/). The statistical significance of difference was calculated using nonparametric one-way ANOVA with Dunnett’s post hoc analysis or Student’s t-test. *P* values of < 0.05 were considered statistically significant.

## Supplementary Information

**Additional file 1: Table S1.** Supplementary table shows the raw data in the manuscript.

**Additional file 2: Figure S1.** PCR amplification of the *zrlA* gene in *A. baumannii* strains. Amplicons of 579 bp were detected in all *A. baumannii* strains tested. **Figure S2.** Production of recombinant ZrlA proteins. SDS-PAGE was performed to detect recombinant proteins of ca. 24 kDa (arrow).

## Data Availability

All data generated or analyzed during this study are included in this published article and its supplementary information files.

## Abbreviations

EVs: Extracellular vesicles
OMV: Outer membrane vesicle
Zn: Zinc
Zur: Zn uptake-regulator
ZrlA: Zur-regulated lipoprotein A
DD-Cpases: D-alanine-D-alanine carboxypeptidases
OPTA: Fluorimetric *o*-phthaldialdehyde
MICs: Minimum inhibitory concentrations
NTA: Nanoparticle tracking analysis
PBPs: Penicillin-binding proteins
HMM: High molecular mass
LMM: Low molecular mass
