# Supplementary material for: The role of Zur-regulated lipoprotein A in bacterial morphology, antimicrobial susceptibility, and production of outer membrane vesicles in Acinetobacter baumannii
Source: BMC Microbiol. 2021 Jan 18;21:27. doi: 10.1186/s12866-020-02083-0 (PMC7812711; doi:10.1186/s12866-020-02083-0)
Supplement: Supplementary file 2 — Additional file 2: Figure S1. PCR amplification of the zrlA gene in A. baumannii strains. Amplicons of 579 bp were detected in all A. baumannii strains tested. Figure S2. Production of recombinant ZrlA proteins. SDS-PAGE was performed to detect recombinant proteins of ca. 24 kDa (arrow). [file 12866_2020_2083_MOESM2_ESM.pptx]

## Slide 1
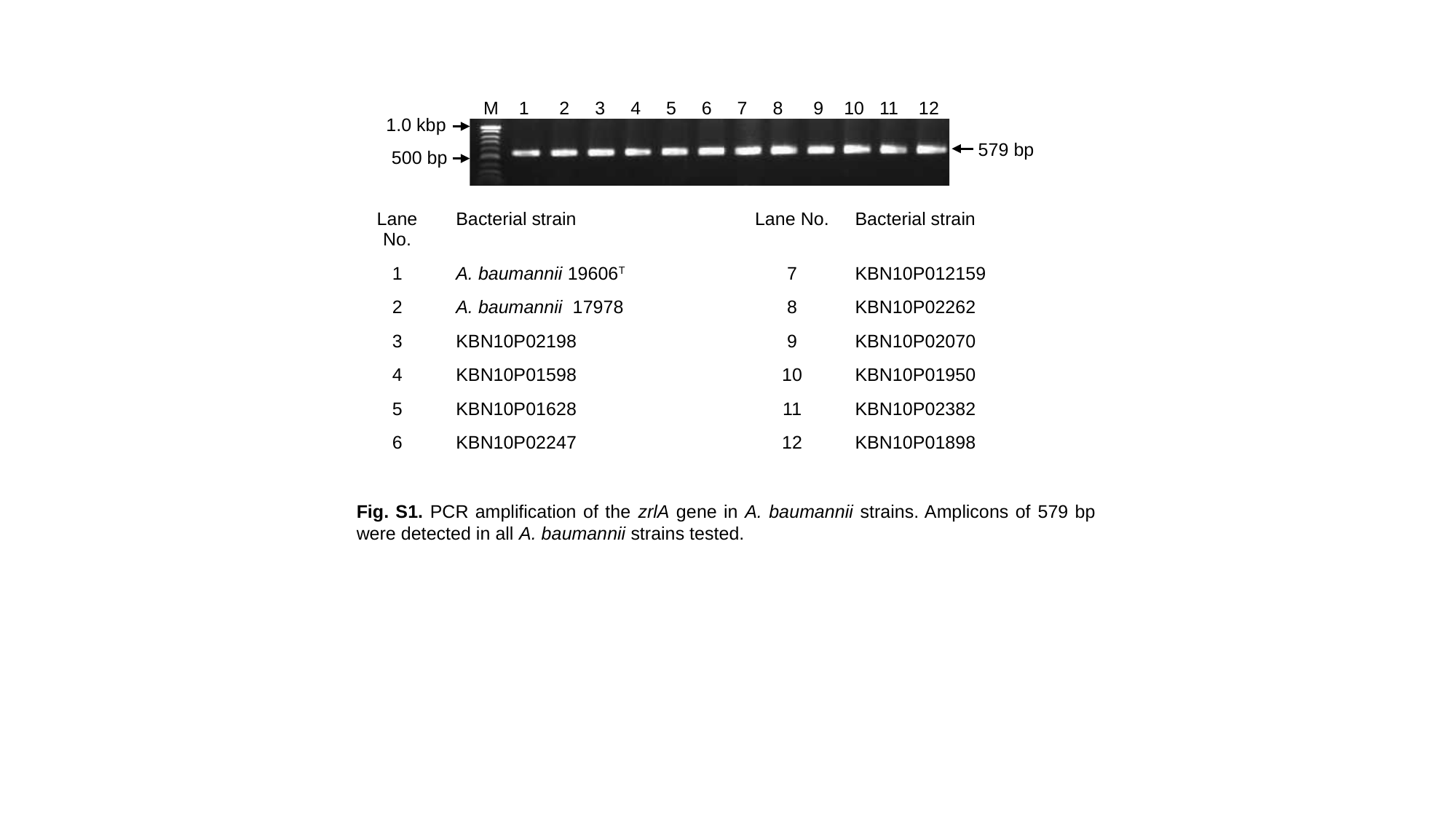

M 1 2 3 4 5 6 7 8 9 10 11 12
1.0 kbp
500 bp
579 bp
| Lane No. | Bacterial strain | Lane No. | Bacterial strain |
| --- | --- | --- | --- |
| 1 | A. baumannii 19606T | 7 | KBN10P012159 |
| 2 | A. baumannii 17978 | 8 | KBN10P02262 |
| 3 | KBN10P02198 | 9 | KBN10P02070 |
| 4 | KBN10P01598 | 10 | KBN10P01950 |
| 5 | KBN10P01628 | 11 | KBN10P02382 |
| 6 | KBN10P02247 | 12 | KBN10P01898 |
Fig. S1. PCR amplification of the zrlA gene in A. baumannii strains. Amplicons of 579 bp were detected in all A. baumannii strains tested.

## Slide 2
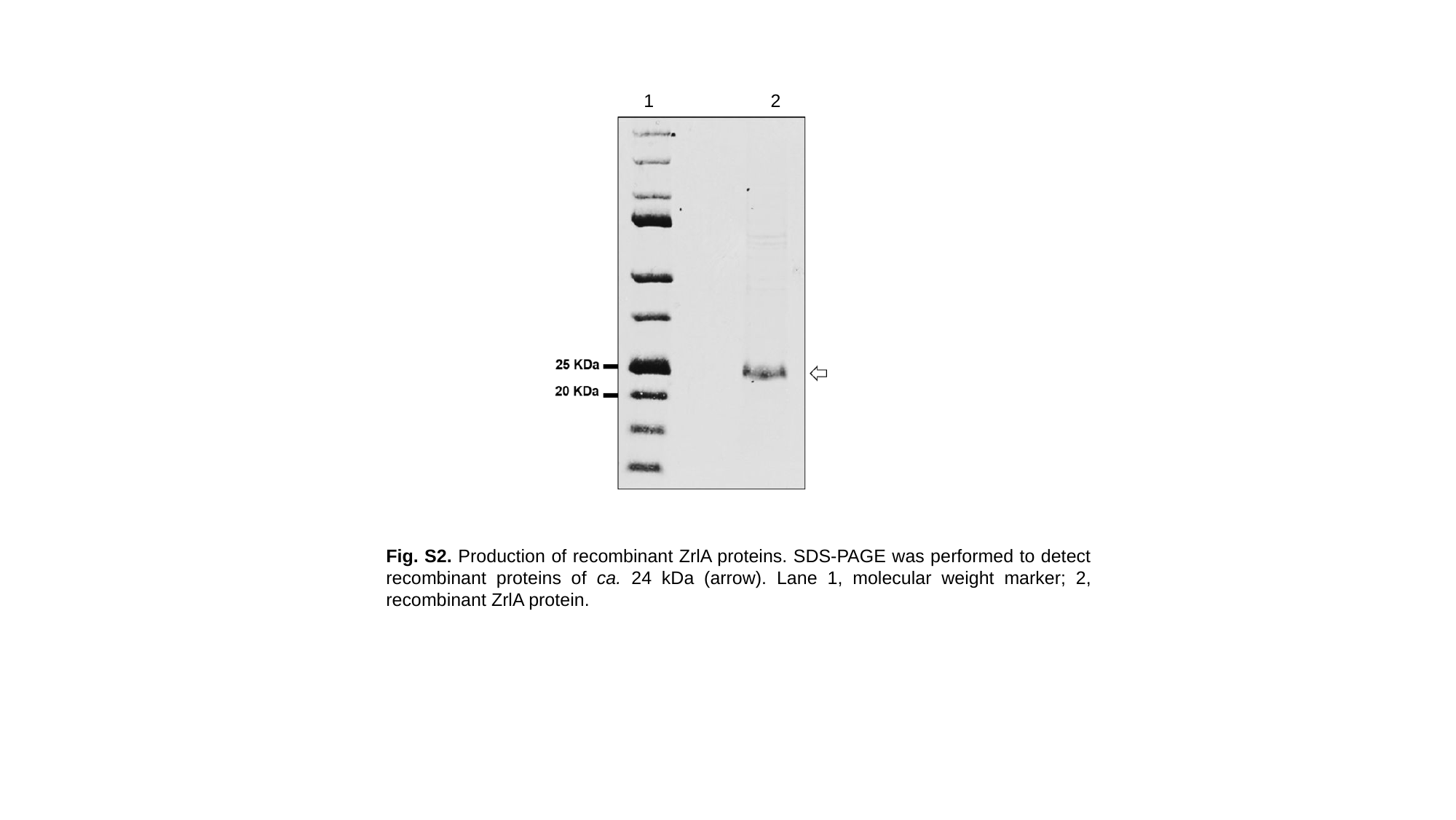

1 2
Fig. S2. Production of recombinant ZrlA proteins. SDS-PAGE was performed to detect recombinant proteins of ca. 24 kDa (arrow). Lane 1, molecular weight marker; 2, recombinant ZrlA protein.
